# Morphometric and Histological Insights to Aid Graft Selection for Anterior Cruciate Ligament Reconstruction

**DOI:** 10.7759/cureus.102899

**Published:** 2026-02-03

**Authors:** Robin Saini, Ritu Sehgal, Arthi Ganapathy, Subrata Basu Ray

**Affiliations:** 1 Department of Anatomy, All India Institute of Medical Sciences, New Delhi, IND

**Keywords:** acl injury, anterior cruciate ligament, anterior cruciate ligament reconstruction (aclr), femoral footprint, graft selection, histology, integrated collagen density, morphometry, the knee joint, tibial footprint

## Abstract

Background: Anterior cruciate ligament (ACL) injuries are among the most common and debilitating knee injuries, especially in athletes. While autografts are widely used for ACL reconstruction (ACLR), the optimal choice of tendon remains controversial. This study aimed to analyze and compare the morphometric and histological characteristics of ACL and periarticular knee tendons (patellar, quadriceps, semitendinosus, gracilis, plantaris), in order to assess their suitability as grafts for ACLR.

Methodology: Thirty knee joints from 15 formalin-embalmed adult cadavers of 12 (80%) males and 3 (20%) females were dissected. Morphometric parameters (length, width, and thickness) of ACL and periarticular tendons were recorded using digital calipers. Femoral and tibial footprints of the ACL were analyzed for size and shape. Histological sections were stained with hematoxylin and eosin (H&E) and Masson’s Trichrome (MT) stains, and the integrated collagen density was quantified using ImageJ software (National Institutes of Health, Bethesda, MD).

Results: Patellar and quadriceps tendons had the largest dimensions, while gracilis showed the highest integrated collagen density, closely resembling that of the ACL. The plantaris tendon, though long, was often absent and structurally less robust. The femoral and tibial footprints displayed variable shapes, with half-moon and droplet patterns being most common, suggesting the need for personalized tunnel placement.

Conclusion: The patellar, quadriceps and gracilis tendons emerge as promising grafts due to favorable morphometry, histology and integrated collagen density. Quadriceps is a viable option for ACLR, while the gracilis with plantaris is an alternative option, depending on the availability of plantaris. This study provides baseline anatomical data to guide graft selection in ACLR and highlights the need for further biomechanical and clinical validation.

## Introduction

The stability of the body’s largest synovial joint, the knee, is maintained by surrounding ligaments, menisci, bursae, and the fibrous capsule, which collectively restrict excessive movement [1]. Injuries of the anterior cruciate ligament (ACL) are relatively frequent, with an estimated incidence of 1/3000 individuals annually, 70% of these being sports-related injuries. Isolated ACL injuries account for nearly 50% of all knee ligament injuries [2,3].

Since ACL plays a crucial role in stabilizing the tibia and femur during flexion-extension and in providing rotary stability during medial/lateral rotation, varus/valgus angulations, and their combinations, ACL injuries lead to knee instability [1,4-7]. Most ACL tears in athletes occur during non-contact pivoting with anterior tibial translation in slight flexion and valgus, and are more common in women [8,9]. ACL reconstruction (ACLR) is the treatment of choice for athletes with complete ACL tears; graft selection plays a key role in the surgical outcome for restoring knee stability. Common grafts include autografts such as bone-patellar tendon-bone (BTB), hamstring (semitendinosus with or without gracilis), quadriceps tendon (with or without bone), and allografts from genetically non-identical human donors [10].

Although numerous studies have evaluated the comparative benefits and risks of various types of ACL grafts, consensus does not exist on the ideal graft choice for reconstruction surgeries. Precise morphometric data on ACL anatomy and fiber thickness may be essential criteria for graft selection, yet such data remain scarce and elusive. This morphometric and histological data may prove immensely useful for knee surgeons. Considering the critical role of ACL in knee stability, its high injury rate among athletes, and the lack of surgical consensus about the ideal choice of graft, this study sought to compare the morphometric and histological characteristics of the ACL and five adjacent periarticular knee tendons to define and standardize anatomical criteria for optimal graft selection for ACLR.

## Materials and methods

Ethical approval was obtained from the Institutional Ethics Committee before dissection, after which the bilateral knee joints of 15 elderly, formalin-embalmed cadavers from North India were dissected. The cadavers used in this study were part of the Voluntary Body Donation Program (regulated by the Anatomy Act of 1949, permitted for research and educational use). Cadavers of both sexes, aged 60-90 years, with no known history of knee joint pathology or surgery, were included in the study.

The sample size was estimated for determining a single population mean for a quantitative variable using the formula:

\[ n = \frac{Z^{2} \times SD^{2}}{d^{2}} \]

ACL morphometric variability reported in an Indian cadaveric study [11] was used as a reference, with a standard deviation (SD) of approximately 2.3 mm for ACL length. At a 95% confidence level (*Z* = 1.96) and assuming a precision (*d*) of 1 mm, the calculated minimum sample size was approximately 21 knees. The present study included 30 knee joints, exceeding the minimum required sample size and thereby providing adequate precision for morphometric estimation. This study utilized 30 knee joints from 15 adult cadavers, which is consistent with sample sizes reported in anatomical and morphometric cadaver research [12,13].

Morphometry

**Figure 1:**
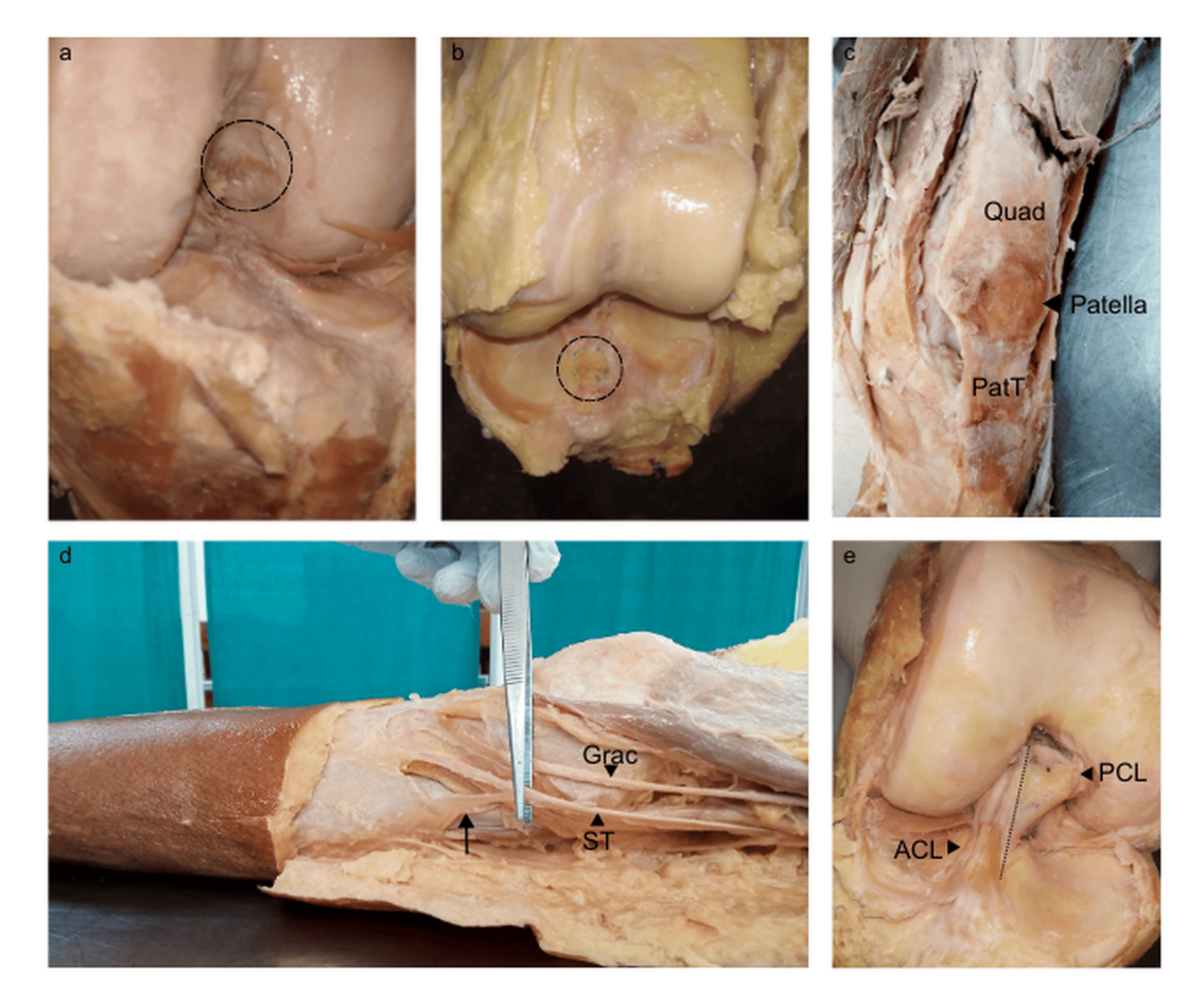
Dissected knee joints showing the attachments of the ACL and periarticular tendons. (a) Femoral footprint; (b) tibial footprint; (c) attachments of the quadriceps (Quad) and patellar (PatT) tendons to the patella (black arrowhead); (d) gracilis (Grac) and semitendinosus (ST) tendons, with the latter showing an aberrant slip merging with the deep fascia of the leg (black arrow). (e) The ACL winds anterolaterally around the PCL, with the two cruciate ligaments demarcated by a dotted line. PCL, posterior cruciate ligament; ACL, anterior cruciate ligament

The knee joints of each cadaver were carefully dissected to expose and identify the following structures: patella anteriorly with quadriceps tendon (Quad) attached proximally and patellar tendon (PatT) attached distally; gracilis (Grac) tendon medially; tendons of semitendinosus (ST) and plantaris (Plant) posteriorly. Dissected knee joints were meticulously examined to trace the ACL to its femoral and tibial attachment sites (footprints), noting its orientation relative to the posterior cruciate ligament (PCL), and the periarticular tendons were traced to their attachments (Figure 1). 

The entire tendons of Grac, ST, and Plant were detached, and sliding digital Vernier calipers (Thermisto TH-M61, 150 mm/6ʺ, precision 0.01 mm; Thermisto, India) were used to record the following morphometric parameters: tendon length (from muscle to bony attachment) and width and thickness measured at the proximal and distal ends of each tendon, as well as at the midpoint between the two ends. The length of the Plant tendon was measured in two or three parts, because its length exceeded the maximum length measurable with the calipers (150 mm). It was measured in two or three parts, depending on whether the length of the tendon was less or more than twice the maximum length measurable. If the tendon length was less than twice the maximum length measurable with the calipers, it was measured in two parts, while if it exceeded twice the maximum length measurable, it was measured in three parts. The same parameters were recorded for the Quad and PatT in situ using digital calipers before detaching the PatT from the tibial tuberosity. Comparison of the width and thickness of each structure at three sites was done in order to understand its structural morphology.

Dissection of the extra-capsular tendons was followed by clearing the anterior fibrous capsule of the knee joint to expose the ACL, using the anterior approach [14,15]. The ACL was cleaned off its synovial sheath and then detached from its tibial and femoral attachments before measuring the same dimensions (length, width, and thickness at proximal and distal ends, and at the midpoint between the two ends) using digital calipers. The maximum and minimum diameters of the tibial footprint (TF) and femoral footprint (FF) were recorded using the calipers, and their shape was noted.

The length of the ACL was compared with the length of the other five tendons around the knee joint; the width and the thickness of the ACL were compared with those of the other five tendons at three sites (proximal, middle & distal). The morphometric data (length, width, and thickness of ACL and the five tendons) collected by dissecting the left and right knee joints and surrounding lower limb regions were compared and analyzed for differences between the left and right sides for each structure.

Histology

Tissue samples were collected from the intermediate part of the ACL and the five tendons and stored in 10% neutral buffered formalin solution [16]. All the samples were processed for paraffin wax embedding, and longitudinal sections were cut at 6 µm using a rotary microtome (Thermo Scientific Microm HM 340 E, Thermo Fisher Scientific, Waltham, MA). The sections were stained with hematoxylin and eosin (H&E) and Masson’s Trichrome (MT) stains. Each stained slide was systematically scanned at 10x magnification, and only the highest-quality sections were selected for image acquisition at 40x magnification. Images were captured using the built-in Nikon Digital Sight 10 camera in conjunction with NIS-Elements AR 6.01.00 imaging software (Nikon Instruments Inc., Tokyo, Japan). Semi-quantitative histological analysis was performed based mainly on three parameters: presence of fibrocartilage, cellularity, and vascularity.

Integrated Density of Collagen Bundles

Images of H&E-stained slides captured at 40x magnification were analyzed using ImageJ software. The scale was first calibrated using a scale bar on the image. All selected images were converted to an 8-bit grayscale format, then image thresholding was applied, and the total image area was calculated. The integrated density of collagen in each image was determined using the following formula: Integrated Density (ID) = mean gray value (0-255) × total image area [17].

Statistical analysis

For statistical analysis and graphical representation of data, the commercially available software GraphPad Prism 10 was used (GraphPad Software, San Diego, CA). To assess the normality of the data, the Kolmogorov-Smirnov test and the Shapiro-Wilk test were employed. Data sets that followed a normal distribution were analyzed using one-way analysis of variance (ANOVA) followed by Dunnett’s multiple comparison test. For data sets that did not follow a normal distribution, the Kruskal-Wallis test or the Wilcoxon signed rank test, along with Dunnett’s multiple comparison test, were used. All data are presented as mean ± SD. One-way ANOVA and Kruskal-Wallis tests, followed by Dunnett’s multiple comparison test, were used to calculate *P*-values for assessing the significance of differences in morphometric measurements (length, width, and thickness) of the ACL and the five tendons, using GraphPad Prism 10. *P*-values were calculated to assess whether the differences in dimensions may be considered non-significant (NS), significant (S), or highly significant (HS).

## Results

The ACL from the right knee of an 85-year-old female cadaver with severe unilateral degeneration was excluded, although the surrounding tendons were retained. Age-related changes, such as cartilage bruising and excess infrapatellar fat, were also observed. The plantaris tendon was congenitally absent bilaterally in three cadavers and unilaterally in another three, allowing its dissection in only 21 knee joints (70%) for morphometric and histological analysis.

Morphometric data

Comparison of Morphometric Data of ACL and Periarticular Tendons

The ACL was wider but thinner at the proximal and distal ends, constricted in the middle where it was thickest (Table 1). The PatT was widest and thickest proximally, gradually narrowing from proximal to distal end, being narrowest and thinnest distally. The Quad tendon was found to be widest and thickest distally, narrowest, and thinnest proximally. The tendons of ST and Grac showed similar trends: width initially decreased slightly from the proximal to the middle portion, then increased significantly toward the distal end. Thickness gradually decreased from the proximal to the distal end along the length of the tendon. The tendon of the vestigial Plant muscle was absent in 40% of subjects (6 of 15; bilateral in 3 and unilateral in 3). It had the longest but narrowest and thinnest tendon among the six structures studied. The tendon was widest and thickest proximally, gradually narrowing and thinning from the proximal to distal end.

**Table 1:** Statistical analysis of morphometric data for ACL and periarticular tendons. PatT, patellar tendon; Quad, quadriceps; ST, semitendinosus; Grac, gracilis; Plant, plantaris; SD, standard deviation; ACL, anterior cruciate ligament

S. no.	Structure	*n* (%)	Mean length ± SD (mm)	Mean width ± SD (mm)			Mean thickness ± SD (mm)		
				Proximal	Middle	Distal	Proximal	Middle	Distal
1	ACL	29 (96.67)	33.61 ± 4.72	13.0 ± 2.3	9.90 ± 1.50	13.0 ± 2.2	2.2 ± 0.89	3.29 ± 1.09	1.5 ± 0.59
2	PatT	30 (100)	44.67 ± 9.09	29.0 ± 2.3	24.64 ± 2.36	22.0 ± 2.8	3.4 ± 0.91	3.11 ± 0.84	2.8 ± 0.99
3	Quad	30 (100)	76.02 ± 12.15	18.0 ± 6.6	27.97 ± 7.13	43.0 ± 4.7	3.3 ± 1.6	5.07 ± 3.11	6.0 ± 1.8
4	ST	30 (100)	132.6 ± 36.48	6.0 ± 1.1	4.45 ± 0.76	10.0 ± 6.6	2.5 ± 0.55	2.17 ± 0.50	1.6 ± 0.45
5	Grac	30 (100)	98.23 ± 31.07	4.4 ± 1.1	3.93 ± 0.83	7.1 ± 4.1	1.8 ± 0.44	1.5 ± 0.38	1.1 ± 0.27
6	Plant	21 (70)	322.3 ± 21.81	3.2 ± 1.2	2.83 ± 1.30	2.8 ± 2.1	0.94 ± 0.77	0.59 ± 0.47	0.67 ± 0.46

The differences in the length of the ACL compared to those of the five tendons were all highly significant. The width differences between ACL and four muscle tendons (PatT, Quad, Grac, and Plant) were also found highly significant, as were the width differences between ACL and ST at the proximal end and in the middle. However, the difference in width between the distal ends of ACL and ST was found to be non-significant. The proximal and distal ends of ACL did not differ significantly in thickness from those of ST and Grac. The ACL did not differ significantly in thickness in the middle from PatT. Other differences in thickness between ACL and muscle tendons were either significant (*P* < 0.05) or highly significant (*P* ≤ 0.0001).

Comparison of Morphometric Data for Left/Right Differences

The length of each left-sided structure (ACL and five knee tendons) was compared with that of the corresponding right-sided structure; the width and thickness of each left-sided structure were compared with those of the corresponding right-sided structure at three sites (proximal, middle, and distal). *P*-values were calculated to assess whether the differences in dimensions may be considered non-significant or significant. Differential comparison of the morphometric data revealed that the differences in dimensions (length, width, and thickness) between the left and right sides were all non-significant. This suggests that the morphometric characteristics of all these structures are consistent bilaterally (Table 2).

**Table 2:** Statistical analysis of morphometric data of left and right ACL and periarticular knee tendons. PatT, patellar tendon; Quad, quadriceps; ST, semitendinosus; Grac, gracilis; Plant, plantaris; Lt., left; Rt., right; SD, standard deviation; ACL, anterior cruciate ligament

Structure	*n* (%)		Mean length ± SD (mm)		Mean width ± SD (mm)						Mean thickness ± SD (mm)					
	Lt.	Rt.	Lt.	Rt.	Proximal		Middle		Distal		Proximal		Middle		Distal	
					Lt.	Rt.	Lt.	Rt.	Lt.	Rt.	Lt.	Rt.	Lt.	Rt.	Lt.	Rt.
ACL	15 (50)	14 (46.67)	33 ± 5.3	35 ± 4	12 ± 2.7	13 ± 1.8	9.9 ± 1.6	9.9 ± 1.5	13 ± 2.2	12 ± 2.2	2.2 ± 0.93	2.2 ± 0.88	3 ± 1.2	3.6 ± 0.95	1.5 ± 0.62	1.6 ± 0.57
PatT	15 (50)	15 (50)	46 ± 10	43 ± 8	29 ± 2.3	28 ± 2.2	25 ± 2.8	25 ± 1.9	22 ± 2.9	23 ± 2.7	3.7 ± 1.0	3.1 ± 0.66	3.1 ± 0.55	3.1 ± 1.1	2.6 ± 0.68	2.9 ± 1.2
Quad	15 (50)	15 (50)	76 ± 11	76 ± 14	19 ± 8.3	16 ± 4.2	27 ± 6	29 ± 8.3	42 ± 4.4	43 ± 5	2.7 ± 1.1	3.8 ± 1.9	4.6 ± 1.7	5.5 ± 1.5	5.6 ± 1.9	6.4 ± 1.8
ST	15 (50)	15 (50)	132 ± 36	133 ± 38	6.0 ±1.2	6.0 ± 1.0	4.5 ± 0.83	4.4 ± 0.73	11 ± 8.5	8.8 ± 3.9	2.5 ± 0.56	2.4 ± 0.55	2.2 ± 0.39	2.2 ± 0.61	1.7 ± 0.52	1.5 ± 0.36
Grac	15 (50)	15 (50)	98 ± 32	99 ± 31	4.3 ± 0.84	4.6 ±1.3	3.9 ± 0.81	4 ± 0.88	7.0 ± 4.1	7.1 ± 4.3	1.7 ± 0.39	1.8 ± 0.49	1.5 ± 0.33	1.5 ± 0.45	1.1 ± 0.29	1.1 ± 0.26
Plant	11 (36.67)	10 (33.33)	318 ± 25	327 ± 18	3.3 ± 1.2	3.1 ± 1.4	3 ± 1.3	2.6 ± 1.3	2.9 ± 2.2	2.6 ± 2	0.83 ± 0.43	1.1 ± 1.0	0.48 ± 0.22	0.72 ± 0.64	0.58 ± 0.28	0.76 ± 0.61

Morphology and Morphometry of FF and TFs of the ACL

**Figure 2:**
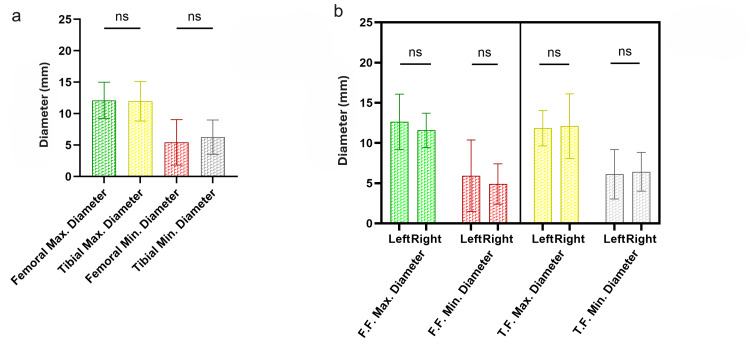
(a) Bar diagrams comparing the maximum and minimum diameters of femoral (FF) and tibial footprints (TF); (b) bar diagrams comparing the left and right maximum and minimum diameters of FF and TF. Panel (a) shows that differences in the maximum and minimum diameters of FF and TF are non-significant (ns) (*P* ≥ 0.05). Panel (b) shows that differences in left and right maximum and minimum diameters of both footprints are ns (*P* ≥ 0.05).

The FF of the ACL showed its widest dimension (maximum diameter) oriented in an oblique anteroposterior direction (mean 12.12 ± 2.88 mm), while its narrowest dimension (minimum diameter) was oriented in an oblique superoinferior direction (mean 5.43 ± 3.61 mm). Analysis of the shape of the FF (Figure 1a) revealed six distinct morphological patterns: half-moon shape, observed in 13 of 29 knee joints (44.83%); comma shape, observed in eight joints (27.59%); quadrilateral and elliptical shapes, observed in three joints each (10.34%); and bean (reniform) and Y shapes, observed in one joint each (3.49%). These findings highlight the numerous variations in the FF morphology, with the half-moon and comma shapes being the predominant patterns (Figure 2).

The TF of the ACL showed its widest dimension (maximum diameter) oriented in a roughly antero-posterior direction (mean 11.96 ± 3.15 mm), while its narrowest dimension (minimum diameter) was oriented in an oblique transverse or medio-lateral direction (mean 6.26 ± 2.72 mm). Analysis of the shape of the TF (Figure 1b) revealed five distinct morphological patterns: droplet (tear) shape, the most common, observed in 14 of 29 knee joints (48.28%); elliptical shape, observed in nine joints (31.03%); spindle shape, observed in three joints (10.34%); quadrilateral shape, observed in two joints (6.9%); and half-moon shape, observed in one joint (3.49%). These findings highlight the numerous variations in the TF morphology, with the droplet and elliptical shapes being the predominant patterns.

Differences in the maximum diameters of FF and TF, as well as their minimum diameters, were found to be statistically non-significant (Figure 2a). Differences in both diameters (between left and right sides) of both footprints (FF and TF) were all found to be non-significant (Figure 2b). These findings highlight the almost equal size of ACL bony attachment sites at either end.

Histological data

**Figure 3:**
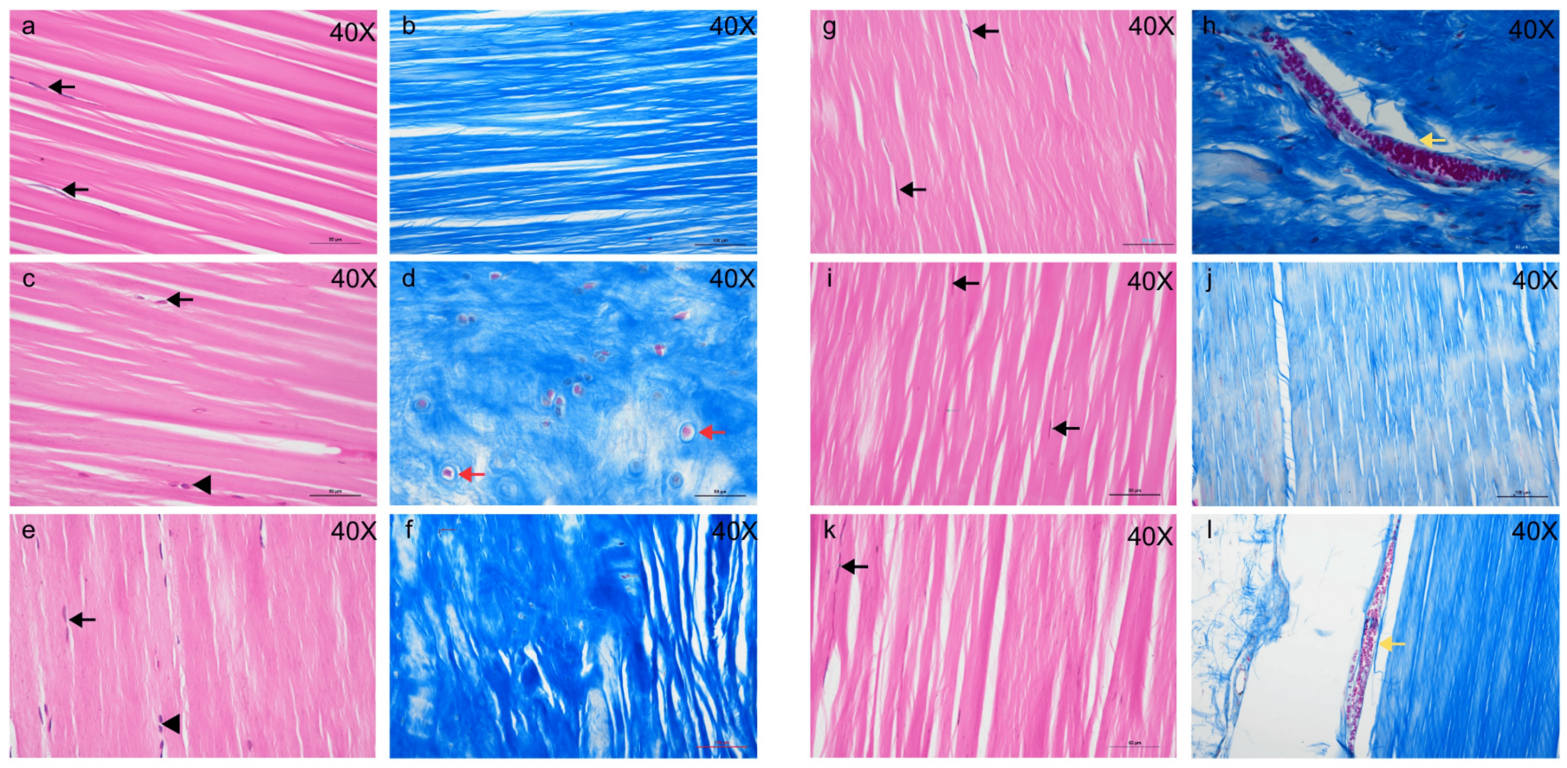
Photomicrographs of H&E-stained (a, c, e, g, i, and k) and MT-stained (b, d, f, h, j, and l) sections of Gracilis (a and b), Quadriceps (c and d), ACL (e and f), Patellar tendon (g and h), Plantaris (i and j), and Semitendinosus (k and l). Regularly arranged, parallel collagen bundles are observed, with numerous interspersed fibroblasts (black arrowheads), fibrocytes (black arrows), and blood vessels (yellow arrows) indicating good vascularity in the tendons. The Quadriceps tendon and ACL show fibrocartilage with wavy collagen bundles and chondrocyte nuclei (red arrows). ACL, anterior cruciate ligament

Examination of the H&E- and MT-stained sections of the intermediate part of the ACL (Figures 3e-3f) revealed densely packed, parallel collagen bundles stained pink (lilac), indicative of the eosinophilic nature of the collagen matrix. The fibers were oriented longitudinally, reflecting the tissue’s unidirectional tensile strength. Elongated, elliptical nuclei of metabolically active fibroblasts were stained blue (lighter violet/purple) with hematoxylin (Figure 3e, black arrowhead), while flattened, more elongated, and heterochromatic nuclei of less active fibrocytes were stained darker blue (deep violet) with hematoxylin (Figure 3e, black arrows) and were interspersed between the collagen fiber bundles. The nuclei were aligned in the direction of the collagen fibers, consistent with the cellular organization of ligaments. The extracellular matrix (ECM) appeared homogeneous and uniform, with narrow, slit-like interstitial spaces between the collagen bundles, occupying a very small percentage of the microscopic field.

A significant finding was the presence of islands of fibrocartilage scattered in patches within the regularly arranged fascicles of dense collagen and cells that characterize the microscopic anatomy of the ACL, suggesting its stress-bearing function and susceptibility to wear-and-tear. Fibrocartilage was identified in H&E-stained sections, but it was more clearly demonstrated in MT-stained sections (Figure 3f). The characteristic features that helped identify the fibrocartilage included individually scattered or linearly aligned chondrocytes with rounded nuclei, occupying distinct lacunae (Figure 3f) within the ECM, between dense, irregularly arranged collagen bundles.

Examination of the H&E- and MT-stained sections of the intermediate parts of the five periarticular knee tendons revealed microscopic features consistent with the cellular organization of tendons, but also remarkably similar to those of the ACL. The extracellular matrix was minimal, with very few metabolically active fibroblasts and fibrocytes (cellularity was lowest in Plant and ST, increasing in PatT, Quad, and Grac, and most numerous in the ACL). The ACL showed the highest cellularity and vascularity among the six structures studied, in terms of the number of fibroblasts, fibrocytes, and blood vessels observed on slide scanning. Numerous islands of fibrocartilage, most abundant in the Quad (Figure 3d, small red arrows) compared to the ACL and the other four tendons, as well as numerous blood vessels (Figures 3h, 3l, yellow arrows), were observed in MT-stained sections. Fibrocartilage was notably absent in tissue sections of ST, Grac, and Plant, while only traces were observed in PatT. Histological data revealed that the ACL is most similar in all qualitative aspects, including cellularity, vascularity, and presence of fibrocartilage, to the patellar tendon and the quadriceps tendon (Table 3).

**Table 3:** Comparison of histological data of ACL and periarticular tendons. +, scanty/low; ++, moderate/medium; +++, abundant/high ACL, anterior cruciate ligament

S. No.	Structure	Fibrocartilage	Cellularity	Vascularity
1	ACL	++	+++	+++
2	PatT	+	++	++
3	Quad	+++	++	+
4	ST	Absent	+	++
5	Grac	Absent	++	+
6	Plant	Absent	+	+

Quantification and Comparison of Integrated Collagen Bundle Density

**Figure 4:**
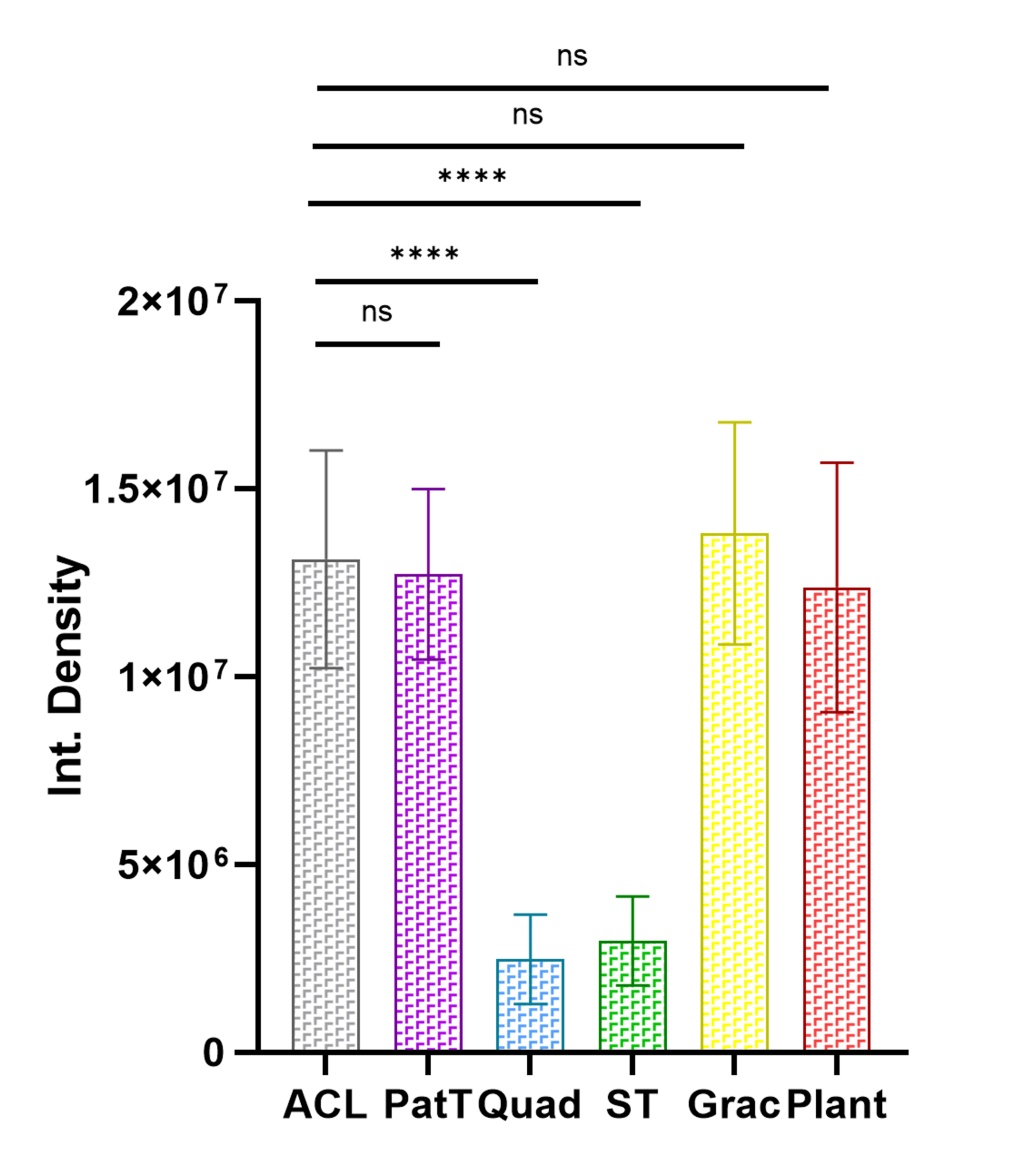
Bar diagram comparing the integrated density (ID) of collagen bundles in the ACL and periarticular tendons. Data graphically represented as mean ± SD; ns, non-significant (*P* ≥ 0.05); **** highly significant (*P* < 0.0001); PatT, patellar tendon; Quad, quadriceps; ST, semitendinosus; Grac, gracilis; Plant, plantaris; ACL, anterior cruciate ligament

The ID values obtained reflect the density and arrangement of collagen bundles, with higher values suggesting denser packing and potentially better alignment (Table 4). The gracilis tendon was found to have the highest mean integrated density, while the quadriceps tendon had the lowest ID value. The semitendinosus and quadriceps tendons showed significantly lower integrated densities than the ACL, patellar, gracilis, and plantaris tendons (HS; *P* < 0.0001). The differences in integrated density between the ACL, patellar, gracilis, and plantaris tendons were all non-significant (NS; *P* ≥ 0.05). The integrated density data suggest that the patellar tendon and the tendons of gracilis and plantaris have ID values closest to those of the ACL (Figure 4).

**Table 4:** Descriptive analysis of integrated density (ID) of collagen bundles. Denominators for % calculations: 29 (ACL), 30 (PatT, Quad, ST, and Grac), 21 (Plant) PatT, patellar tendon; Quad, quadriceps; ST, semitendinosus; Grac, gracilis; Plant, plantaris; SD, standard deviation; ACL, anterior cruciate ligament

S. no.	Structure	*n* (%)	Mean integrated density (ID)	SD	Range (Maximum – Minimum value)
1	ACL	20 (68.97)	13124741	2901617	7287536
2	PatT	19 (63.33)	12721660	2271308	6628194
3	Quad	22 (73.33)	2478152	1189214	3805987
4	ST	25 (83.33)	2961468	1182331	4151930
5	Grac	22 (73.33)	13808069	2957884	7026977
6	Plant	19 (90.48)	12371170	3317350	13265829

## Discussion

ACL grafts that are commonly employed include ST (with or without Grac), Quad, and Pat T [10,18-20]. This study offers morphometric and histological data for aiding the choice of ACL graft. In our study, the morphometry of the Quad (mean length 76.02 ± 12.15 mm) is comparable to that reported by Xerogeanes et al. (mean length 81.1 ± 10.6 mm) [21]. Xerogeanes et al. [21] and Latiff and Olateju [22] have proposed the quadriceps tendon as a morphologically abundant and superior graft choice compared with other tendons. Morphometric and histological evaluation of the quadriceps tendon in our study has also emphasized its viability as a potentially ideal graft. However, the ID value of collagen bundles in the quadriceps tendon was found to be significantly lower than that of the ACL. This may be due to age-related degenerative changes in the Quad tendons of the elderly body donors included in our study. The Quad tendons of young athletic individuals may have higher ID values.

The ST is also commonly used in ACLR surgeries, but previous studies have indicated that, when used alone, it may not be sufficient for ACLR [10]. Gracilis is often harvested alongside ST to compensate, which may lead to functional loss or delayed recovery, adversely affecting its suitability as a graft option. The mean length of the Grac tendon measured in our study (98.23 ± 31.07 mm) was comparable to the observations of Assi et al. (mean length 96 ± 26mm) [23]. The histological evaluation of the Grac tendon revealed that the ID of its collagen bundles is quite similar to that of ACL, indicating that the Grac tendon has the potential to serve as a primary graft option for ACLR, with supplementation with other tendons. Plantaris may serve as a supporting graft (depending on its availability).

The PatT (BTB autograft/allograft) is widely used for ACLR due to its strength and bone-to-bone healing properties. The morphometric measurements of the PatT recorded in the present study (mean length 44.67 ± 9.09 mm), along with its histological structure and its collagen ID value, are comparable to those of ACL, which makes it a suitable graft option in all respects. However, previous studies have shown increased risk of anterior knee pain and functional loss [24-26]. In this study, the Plantaris tendon has been investigated as a graft option in ACLR. Nayak et al. [27] reported morphometric values comparable to those recorded in the present study. Owing to its smaller dimensions, it is unsuitable as a standalone ACL graft; the ID of its collagen bundles closely resembles that of ACL. Being a vestigial muscle with negligible functional loss on removal, it may serve as a useful supplementary graft.

This study acknowledges that knowledge of the accurate dimensions and morphology of FF and TF enables knee surgeons to replicate the native attachment sites of ACL more precisely. Misplacement of the graft could lead to graft failure, rotational instability, and poor functional outcomes. Our findings indicate that the femoral footprint is relatively longer but narrower, while the tibial footprint is shorter and wider, although the differences in maximum and minimum diameters between the two footprints are non-significant. The greater variability in the shape of FF may necessitate patient-specific tunnel placement, whereas the more consistent droplet shape of TF makes for a more predictable graft fixation. Morales-Avalos et al. [28,29] reported semicircular FF and oval TF as the commonest shapes observed, which are quite similar to the half-moon-shaped FF and droplet (tear) shaped TF seen in our study.

## Conclusions

The comprehensive morphometric and histological evaluation in our study provides crucial anatomical baseline data for knee surgeons, which may aid in selecting the most suitable tendon for ACLR. The study offers valuable insights into the structural and histological features of the ACL and five surrounding tendons. The high collagen ID values recorded for PatT and Grac tendons make them attractive graft options. Morphometrically and histologically, Quad and PatT were found to be the best candidates. Despite its length, the vestigial nature of the Plant tendon makes it suitable only as an adjunct graft. Our findings based on morphometry and histology favor the use of the quadriceps tendon as a good graft option, with the patellar tendon a close second. Other viable options would be the use of the gracilis tendon as a primary graft with supplementation with plantaris (if present), or the semitendinosus tendon as a supporting graft. Analysis of the femoral and tibial footprints revealed significant shape differences, indicating the need for personalized graft placement. No notable bilateral differences were observed, suggesting that either limb may be used for graft harvesting. These findings underscore the importance of morphometric and histological factors in graft selection for ACLR.

The limitations of this study relate to the use of formalin-fixed and elderly cadaveric specimens; the morphometric values may differ slightly from those in vivo and in younger, more athletic individuals. Assessment of biomechanical properties like tensile strength in future studies may lend further credibility to graft selection decisions. The middle thickest part of the ACL was selected for histological assessment because this part yielded optimum samples for histological study. Since this study was conducted on the ACL of knee joints dissected from donated cadavers (received by the Anatomy department) of deceased elderly subjects, degenerative changes were noted close to the proximal and distal attachment sites of the ligament in some specimens. A histological and biomechanical study of ACL on fresh cadavers of recently deceased young subjects obtained from the mortuary of the Forensic department may be contemplated in the future.
